# Outcome of a Simple Novel Technique to Reduce Soft Tissue Complications in Open Tendoachilles Injury: A Series of 20 Patients

**DOI:** 10.5704/MOJ.2307.008

**Published:** 2023-07

**Authors:** J Mohd, NA Bhat, ZA Lone, TA Bhat, T Afzal, B Dev, MF Butt, S Gupta

**Affiliations:** 1 Department of Orthopaedics, Government Medical College Anantnag, Anantnag, India; 2 Department of Orthopaedics, Government Medical College Jammu, Jammu, India

**Keywords:** open tendoachilles injury, lavatory pan toilet injury, primary end to end repair, AOFAS hindfoot score

## Abstract

**Introduction:**

Open tendoachilles injuries are rare and associated with significant soft tissues complications. The objective of the present study was to assess the clinical outcome and safety of a simple and minimally invasive technique, with a goal to assess if it may help minimise flap and wound related complications in open tendoachilles injuries.

**Materials and methods:**

This prospective study of four years duration included 20 patients with open tendoachilles injuries managed with a simple minimally invasive tunnel technique. The primary outcome variable was occurrence of a major soft tissue complication. The secondary outcome variables included functional outcome measured using AOFAS Ankle hind foot score, re-rupture of tendoachilles and need for revision surgery.

**Results:**

None of the patients in the present series developed a serious soft tissue complication. Based upon the AOFAS hind foot scoring system, good to excellent outcome was achieved in 19 (95%) patients. All the patients were able to perform tip toe walking at six months post-surgery. None of the patients had a re-rupture of the tendoachilles and no patient needed a revision surgery. The complications encountered include thickening of the tendon at the repair site (15%), superficial wound infection (5%), stitch granuloma (5%) and hypertrophic scar (5%).

**Conclusion:**

This technique seems to be promising in reducing the soft tissue complications associated with the surgical management of open tendoachilles injuries. Most patients had a good final clinical outcome. The technique is safe, simple and reproducible. However, further randomised control studies with a larger sample size assessing the technique are recommended.

## Introduction

Tendoachilles injury is fairly common^1^, even though it is one of the thickest and the stoutest tendons in the human body^2^. Most of the existing literature regarding the tendoachilles injuries pertains to the closed ones and very little literature exists on open injuries^3,4^. The standard approach to management of the open tendoachilles injuries involves adequate wound debridement along with copious saline wash followed by primary repair of the tendon and tension free skin closure^5^. Significant flap and wound related complications have been reported by previous researchers along with a need for revision surgery^2,3,5^. These complications occur due to interference with the peroneal and the posterior tibial angiosomes^6,7^ during placement of extrapolated skin incisions from the edges of the traumatic wound and in part due to undue stretching of the flap during retraction by metallic retractors. Various limited open techniques for closed tendoachilles injuries may be cited^8-11^.

However, no such technique for open cuts of tendoachilles is documented. The objective of the present study was to assess the clinical outcome and safety of a simple and minimally invasive technique, with a goal to assess if it may help minimise flap and wound related complications in open tendoachilles injuries by avoiding undue interference with the skin flaps and angiosomes.

## Materials and Methods

This prospective observational study of four years duration, involved 22 adult patients with unilateral open tendoachilles injury who presented to the Orthopaedic emergency department. The study was completed by 20 patients and the two patients lost to follow-up were excluded from the study. Informed written consent was taken from all the study participants and due approval was taken from the institutional ethical committee (GMCA/IEC/2018/25 dated: 21-03-2018).

Patients with isolated, open, complete tendoachilles cut were included in the study. All patients with closed tendoachilles injury, partial tendoachilles cuts, associated neurovascular or bony injuries, uncontrolled diabetes or peripheral arterial disease and polytrauma were excluded.

The patients were received in the emergency and stabilised using standard Advanced Trauma Life Support (ATLS) protocol. Distal neurovascular status was ascertained. The wound was irrigated with normal saline and prophylactic intravenous antibiotics were administered. Intravenous antibiotic cocktail consisted of Cefuroxime 1.5gm, Amikacin 500mg and Metronidazole 500mg, in accordance with the hospital antibiotic policy. The wound was dressed and adequate analgesia was provided. Radiographs were ordered to rule out any bony injuries.

**Fig 1:**
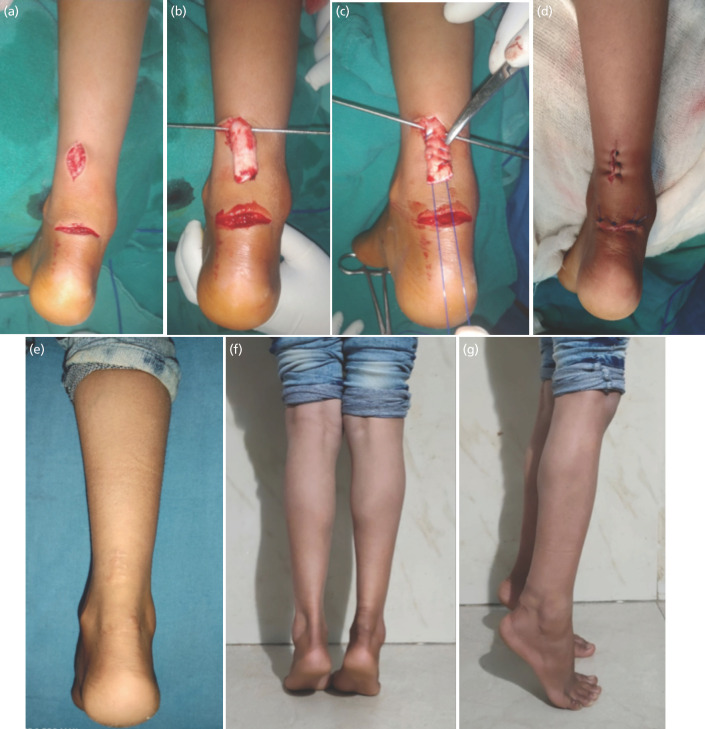
Photographs of a 25 year old female with Indian type Lavatory pan toilet injury who had excellent final result. (a) A midline longitudinal skin incision was made over the tendoachilles, such that the distal extent of the incision was just proximal to the proximal cut end of the tendon. (b) The proximal end of the tendon delivered through the incision and held by a Kirschner wire. (c) The delivered end sutured by Krackow’s technique using PDS II suture. (d) Wounds closed back after primary tendon repair. (e) Good healing of wound at final follow-up. (f, g) Patient able to perform tip toe walking at one year post-surgery.

**Fig 2:**
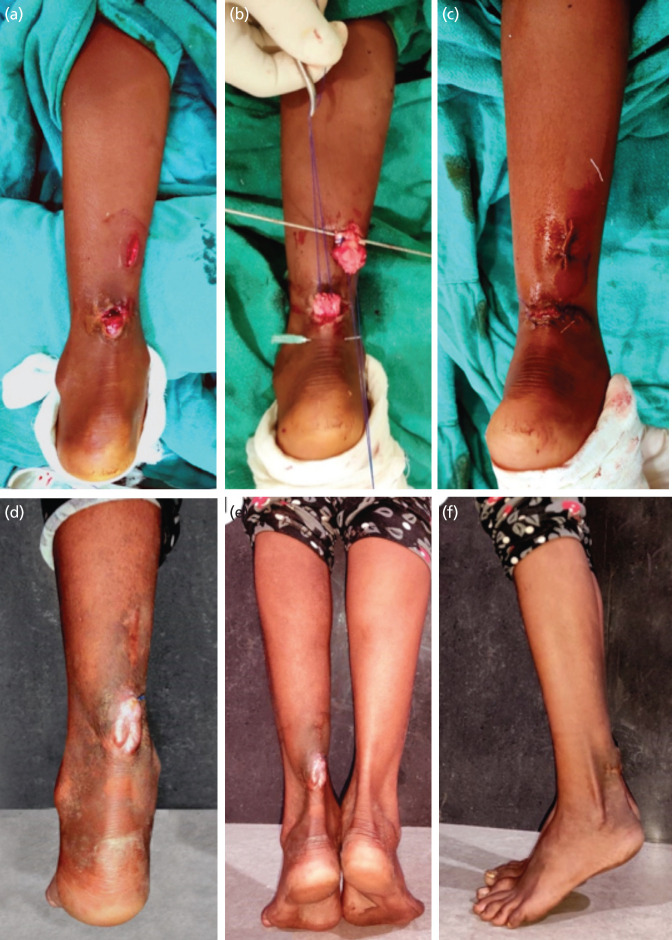
A 26-year-old female presented with two weeks old open tendoachilles cut caused by an agricultural field injury. (a) Proximal longitudinal incion intended to deliver out the proximal end of the tendon. (b) Proximal and the distal ends of the tendon held with proline sutures using Krackow’s tenchnique. (c) Skin closure done after primary tendon repair. (d) Hypertrophic scar at traumatic wound site and a stitch granuloma around a protruding Prolene thread can be appreciated. (e,f) Patient able to perform tip toe walk at final follow-up.

**Fig 3:**
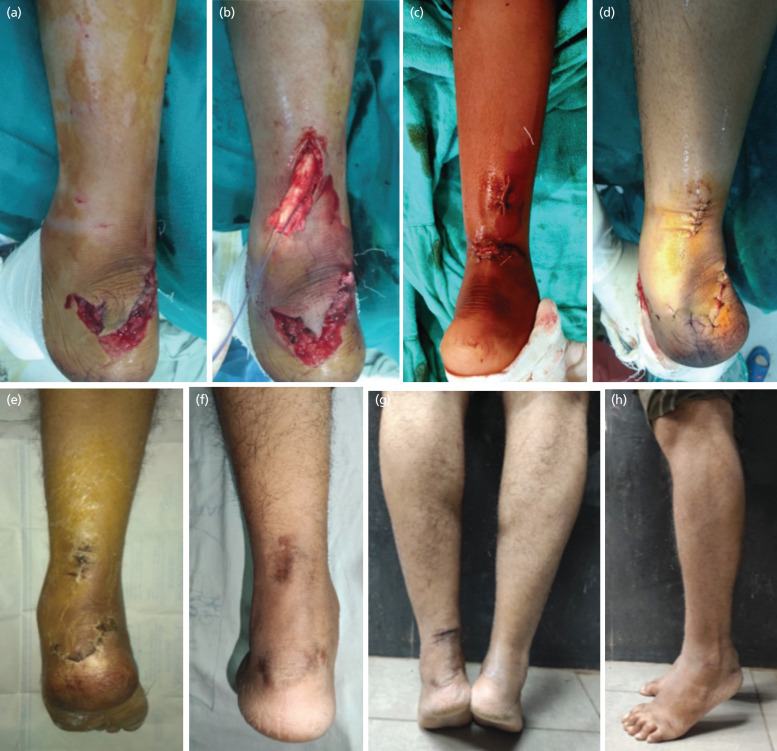
(a) Intra-operative photograph of a 48-year-old male with road traffic accident with open tendoachilles cut, (b,c) proximal end of the tendoachilles being delivered through the midline incision and held using Krackow’s technique, (d) after tendoachilles repair, the skin closed using interrupted sutures. (e) The patient developed superficial wound infection which was managed with antiseptic dresssings and antibiotics. (f) Final wound healing at two years. (g, h) Patient able to perform tip toe walking at final follow-up.

Three surgeons operated all the patients included in the study. All the three surgeons were of comparable competence with same level of experience. All the patients, except one, were operated within 24 hours of injury. The only patient with delayed presentation was a young female who was operated two weeks after open tendoachilles cut. All the patients were operated under spinal anaesthesia in the emergency operating room. Tourniquet was not used and the patients were placed in a prone position. Copious saline irrigation and gross decontamination was followed marginal wound debridement. The proximal cut end of the tendon, which was found to be cranially retracted, was palpated. A midline, two to three centimeter, longitudinal skin incision was made over the tendon, such that the distal extent of the incision was just proximal to the proximal cut end of the tendon (Fig. 1). The skin incision was carried through to the subcutaneous tissue and the tendon sheath. The proximal end of the tendon was delivered through the incision and held by a Kirschner wire (K wire) (Fig. 1 and Fig. 2). The delivered end was sutured using Krackow’s technique (Fig. 1 and Fig. 2) and a mosquito artery forceps was passed via the traumatic laceration through the tunnel and the suture ends were held with the forceps. The proximal end of the tendon was then gently slid back into the tunnel by pulling the suture ends distally. Using gentle skin retraction with foot placed in maximum planter flexion, distal cut end of the tendoachilles was visualised. The distal end of the tendon was then sutured using Krackow’s technique. Two ends of the tendon were approximated by gently pulling upon the suture ends attached to the cut ends of the tendon while placing the knee in flexion and the foot in maximum planter-flexion. Primary end to end tendon repair was completed and a tension free closure of the skin was done using mono-filamentous non-absorbable sutures (Fig. 1 and Fig. 3). We used three types of suture materials for tendoachilles repair. Prolene [Johnson and Johnson, Aurangabad, India] and Ethibond [Johnson and Johnson, Mumbai, India] were used in one patient each. PDS II [Johnson and Johnson, Aurangabad, India] was used in rest of the 18 patients.

All patients were placed in a below knee plaster cast post-surgery with the foot in gravity equinus. Intravenous antibiotics were given for three days. At two weeks, the cast was opened and skin sutures were removed. The patient was placed in another below knee plaster cast for four weeks. At six weeks post-operatively, the plaster cast was changed and the foot was placed in a plantigrade position. The patient was allowed to bear weight on a walking cast, six to eight weeks post-surgery. After eight weeks post-surgery, the patient was started on ankle physiotherapy and a dorsal plaster slab was given to restrict dorsiflexion during walking and prevent rerupture. The patient was allowed to participate in contact sports only after six months post-surgery.

The patients were followed at two weeks, six weeks, three months, six months, one year and two years. At each follow-up the patient was evaluated for any complications. The minimum final follow-up was two years. The primary outcome variable was occurrence of a major soft tissue complication (deep infection, flap necrosis, extensive soft tissue necrosis, etc.). The secondary outcome variables included functional outcome measured by The American Orthopaedic Foot and Ankle Society (AOFAS) Ankle hind foot score^12^, re-rupture of tendoachilles and need for revision surgery. SPSS statistics programme version 20 [IBM, Armonk, NY, USA] was used to carry out the statistical analysis of the data.

## Results

None of the patients in the present series developed a serious soft tissue complication. With regard to clinical outcomes, the mean AOFAS ankle hindfoot score at two years follow-up was 88.25±6.37. Based upon the AOFAS hind foot scoring system, good to excellent outcome was achieved in 19 (95%) patients and none of the patients had a poor outcome at final follow-up. All the patients were able to perform tip toe walking at six months post-surgery. None of the patients had a re-rupture of the tendoachilles and no patient needed a revision surgery.

The patient data, demographic details and results have been summarised in Table I and Table II. Open tendoachilles injuries were found to be 2.3 times more common in males

**Table I:** Patient profile, outcome and complications

S. No.	Age (Years)	Gender	Cause	Side	Operative time (Minutes)	Complications	AOFAS AHS	Final Outcome
1		Male	RTA	Right	35	Superficial wound infection	84	Good
2	30	Male	ASSW	Right	45	None	87	Good
3	19	Male	INLPTI	Right	40	None	91	Excellent
4	26	Female	RTA	Left	30	None	88	Good
5	22	Male	RTA	Right	45	None	93	Excellent
6	33	Male	INLPTI	Right	40	None	82	Good
7	27	Female	AGFI	Right	35	Thickening at tendon repair site	84	Good
8	23	Male	RTA	Left	40	None	91	Excellent
9	40	Male	INLPTI	Right	45	None	93	Excellent
10	26	Female	AGFI	Left	60	Hypertrophic scar, Stitch granuloma	70	Fair
11	20	Male	ASSW	Right	45	None	96	Excellent
12	41	Female	AGFI	Left	35	None	93	Excellent
13	37	Male	RTA	Right	45	Thickening at tendon repair site	84	Good
14	22	Female	INLPTI	Right	50	None	82	Good
15	24	Male	RTA	Left	35	None	89	Good
16	32	Male	RTA	Right	40	None	96	Excellent
17	46	Male	INLPTI	Right	45	Thickening at tendon repair site	84	Good
18	23	Male	AGFI	Left	55	None	96	Excellent
19	31	Male	RTA	Right	45	None	89	Good
20	25	Female	INLPTI	Left	30	None	93	Excellent
**Mean±SD**		**29.75±8.62**				**42±7.67**		**88.25±6.37**

Abbreviations - RTA: Road traffic accident, ASSW: Assault with sharp weapon, INLPTI: Indian lavatory pan toilet injury, AGFI: Agricultural field injury, AOFAS AHS: American Orthopaedic Foot and Ankle Society Ankle hind foot score, SD: Standard deviation

**Table II:** Demographic details of the study participants and functional result achieved (n=20)

Variable	Observation
1. Age of patients (Mean±SD)	(29.75±8.62) years {Range:19-48 years}
2. Gender	
Male	14 patients (70%)
Female	06 patients (30%)
Male : Female	2.3 : 1
3. Mechanism of Injury	
Road Traffic Accident	08 patients (40%)
Indian Lavatory Pan Toilet injury	06 patients (30%)
Agricultural field injury	04 patients (20%)
Assault with sharp weapon	02 patients (10%)
4. Laterality	
Right side	13 patients (65%)
Left side	07 patients (35%)
5. Operative time (Mean±SD)	(42±7.67) minutes
6. AOFAS Ankle hindfoot score (Mean±SD)	88.25±6.37
7. Functional result at two years using AOFAS-AHS	
Excellent	09 (45%) patients
Good	10 (50%) patients
Fair	01 (5%) patient
Poor	0 (0%) patients

Abbreviations - SD: Standard deviation, AOFAS-AHS: American Orthopaedic Foot and Ankle Society Ankle hind foot score

in the present study. Most of the patients were young, with a mean age of 29.75±8.62 (range: 19-48 years). Road traffic accident was the most common mode of injury, constituting 40% of all cases and right side was injured in 65% of the patients. The mean operative time for the surgery was 42±7.67 minutes.

The complications observed and the treatment provided are tabulated in Table III. Thickening of the tendon at repair site, observed in three patients (15%), was the most common complication. Superficial wound infection occurred in one patient in whom Ethibond [Johnson and Johnson, Mumbai, India] was used for tendoachilles repair. Prolene [Johnson and Johnson, Aurangabad, India] was used in one patient for tendoachilles repair, who later developed a stitch granuloma.

**Table III:** Complications encountered during study along with the treatment provided

Complications	Number of patients	Management
Thickening of tendon at repair site	03 (15%)	Conservative (Didn’t bother the patients)
Superficial wound infection	01 (5%)	Conservative (Anti-septic dressings and antibiotics)
Stitch granuloma	01 (5%)	Removal of proline stitch at 7 months
Hypertrophic scar	01 (5%)	Local steroid injection (No benefit)

## Discussion

In the present series, no patient developed a serious soft tissue complication and the final outcome at one year follow-up was good to excellent in 95% of the patients, using AOFAS hind foot score. All the patients were able to perform tip toe walking at six months post-surgery and all the patients were satisfied with the final outcome except for the one female patient with fair outcome who had developed a hypertrophic scar and some restriction in the hindfoot motion. The mean AOFAS hindfoot score at two years follow-up was 88.25±6.37. The final outcome scores observed were comparable to the previous studies^2,3,5^, however the observed wound and soft tissue complications seem to be greatly reduced.

The literature focusing on open tendoachilles injuries is very limited, since closed injuries are much more common worldwide^13,14^. However, there is a significant burden of open tendoachilles lacerations in the developing countries^3,13^. There is a lot of variation in the mechanism of open tendoachilles injuries in existing literature and it seems to differ with geographical location^3^. Road traffic accident, bicycle spoke injury, lavatory pan toilet injury, lawn mower injury and lacerations with broken glass bottles have been reported as various modes of this injury from different corners of the world^2,3,5,15,16^. In the present study, road traffic accident was the most common cause of injury, accounting for 40% of the patients. Indian lavatory pan toilet injury was another significant cause, observed in 30% of the patients.

Open injuries of the tendoachilles predominantly involve the young males. The mean age, at the time of injury, in the present study was 29.75±8.62 years (range:19-48 years). 70% of the patients were males and 65% of the patients were aged between 18-31 years. The observations are similar to Mohsin *et al*^5^, Baindoor *et al*^13^ and Said *et al*^14^, who observed an average age of 34.2 years (range:22-52 years), 35 years (range:18-35 years) and 34 years (range: 18-52 years) at presentation, respectively.

The proximal end of the tendon tends to retract cranially after complete open laceration of tendoachilles. For better visualisation of the proximal end of the tendon during repair, it is imperative to either extend the traumatic laceration at the corner or to use forceful retraction by metallic retractors. However, these add to the risk of skin flap necrosis, infection and other wound related complications^2,3,5^. Dar *et al*^3^, in a series of 12 patients with open lacerations of tendoachilles caused by Indian lavatory pan injury, reported deep infection and sloughing of skin in two patients (16%). Both of the patients were not satisfied with the final result and needed multiple surgeries including a plastic surgery intervention. Mohsin *et al*^5^ observed severe wound infection with extensive soft tissue necrosis and need for second surgery among 11.5% of patients in a study comprising of 26 cases of open tendoachilles injuries. Such complications are attributed to the interference with the angiosomes of the foot and ankle, which tends to occur due to extrapolation or extension of traumatic skin lacerations and due to the forceful retraction of soft tissues^6^.

The tendoachilles derives most of its blood supply from the anterior paratenon which is supplied predominantly by the posterior tibial artery^17,18^. To reduce the wound and skin flap related complications it would be advisable to avoid extension of traumatic lacerations and forceful stretching of skin during retraction to minimise interference with the angiosomes of foot and ankle. Attinger *et al*^6^ recommend a midline skin incision over the tendoachilles that divides the peroneal angiosome from the posterior tibial angiosome. For this purpose, we utilised a minimally invasive technique which utilises a two to three centimeter single longitudinal incision in the midline over the tendoachilles just proximal to the retracted cranial end of the tendon.

In comparison to the previous series, the soft tissue complications seem to be reduced to a great extent^2,3,5^. None of the patients in the present series developed deep infections, flap necrosis, wound dehiscence, re-rupture and no patient needed a revision surgery. The most common complication encountered was thickening of the tendon at repair site which was seen in three (15%) patients.

All the three patients were not bothered by the thickening and hence no intervention was performed. Superficial wound infection was observed in one patient which was managed successfully with antiseptic dressings and antibiotics. There has been a debate regarding the preferred sutural material for repair of tendoachilles^19^. However, PDS II [Johnson and Johnson, Aurangabad, India] has been reported to have lower incidence of infection and better functional outcome when compared with non-absorbable sutures^20^. In the present series, a 26-year-old female, who presented two weeks after the injury, needed removal of Prolene [Johnson and Johnson, Aurangabad, India] for a stitch granuloma at seven months post-surgery. A hypertrophic scar was observed in the same patient at the traumatic laceration site at one year follow-up. Local injection of steroid for the hypertrophic scar did not benefit the patient. Ethibond [Johnson and Johnson, Mumbai, India] was used for tendoachilles repair in one patient and the patient developed superficial wound infection which responded well to antiseptic dressings and antibiotics.

A small sample size and lack of a control group are obvious limitations of the present series. Hence, we recommend large multi-centre randomised control studies to be carried out on this technique.

## Conclusion

This technique seems to be promising in reducing the soft tissue complications associated with the surgical management of open tendoachilles injuries. Most patients had a good final clinical outcome. The technique is safe, simple and reproducible. However, further randomised control studies with a larger sample sizes assessing the technique are recommended.
